# 1353. Safety of AZD7442 (Tixagevimab/Cilgavimab) for Post-exposure Prophylaxis of COVID-19: Final Analysis of the STORM CHASER Phase 3 Study

**DOI:** 10.1093/ofid/ofad500.1190

**Published:** 2023-11-27

**Authors:** Myron J Levin, Andrew Ustianowski, Jesse Thissen, Seth Seegobin, Rohini Beavon, Kanika Dey, Elizabeth J Kelly, Karen A Near, Katie Streicher, Alexandre Kiazand, Mark T Esser

**Affiliations:** University of Colorado Denver School of Medicine, Aurora, Colorado; North Manchester General Hospital, Manchester, England, United Kingdom; AstraZeneca, Cambridge, England, United Kingdom; AstraZeneca, Cambridge, England, United Kingdom; AstraZeneca, Cambridge, England, United Kingdom; AstraZeneca, Cambridge, England, United Kingdom; AstraZeneca, Cambridge, England, United Kingdom; AstraZeneca, Cambridge, England, United Kingdom; AstraZeneca, Cambridge, England, United Kingdom; AstraZeneca, Cambridge, England, United Kingdom; AstraZeneca, Cambridge, England, United Kingdom

## Abstract

**Background:**

In the STORM CHASER phase 3 post-exposure prophylaxis study, 300-mg intramuscular (IM) AZD7442 (tixagevimab/cilgavimab) reduced symptomatic COVID-19 by 33.3% vs placebo at primary analysis (*P*=0.212; median follow-up: 183 days) and was well tolerated. We report final safety data from STORM CHASER.

**Methods:**

In STORM CHASER (NCT04625972), adults without prior SARS-CoV-2 infection or COVID-19 vaccination were enrolled within 8 days of exposure to a SARS-CoV-2–infected individual and randomized 2:1 to receive a single intramuscular dose of 300-mg AZD7442 (N=749) or placebo (N=372). Results are reported from the November 12, 2022 final data cut-off. The primary safety endpoint was assessment of adverse events (AEs), serious adverse events (SAEs), medically attended AEs (MAAEs), and AEs of special interest (AESIs). AESIs included injection site and hypersensitivity reactions. Efficacy data have previously been reported.

**Results:**

Across both the AZD7442 and placebo groups, 928 (82.1%) participants completed the study. Median follow-up was 455 days (∼15 months) in both groups. AEs occurred in 46.5% and 51.9% of participants administered AZD7442 and placebo, respectively (**Table**). Most AEs were mild to moderate in severity; 30 (4.0%) and 26 (7.0%) of participants in the AZD7442 and placebo groups, respectively, reported an AE of grade 3 (severe) or higher. SAEs occurred in 2.7% and 4.3% of AZD7442 and placebo participants, MAAEs in 12.7% and 14.0%, AESIs in 0.5% and 1.1%, and deaths in 0.4% and 0.5%, respectively.

**Figure:**
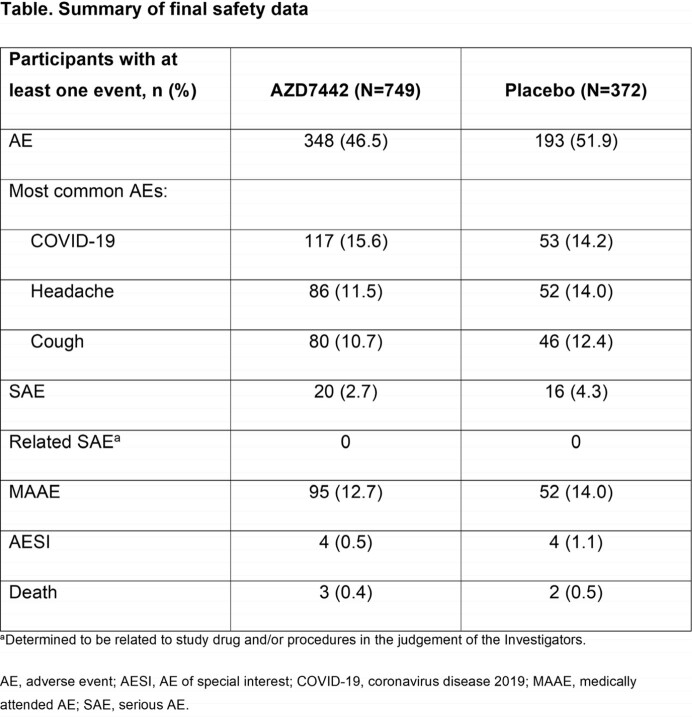

**Conclusion:**

These findings support the long-term safety of AZD7442.

**Disclosures:**

**Myron J. Levin, MD**, Dynavax: Advisor/Consultant|GSK: Advisor/Consultant|GSK: Grant/Research Support|GSK: Data safety monitoring/Advisory board|Johnson & Johnson: Grant/Research Support|Merck & Co.: Advisor/Consultant|Moderna: Grant/Research Support|Novavax: Grant/Research Support|Pfizer: Advisor/Consultant|Seqirus: Advisor/Consultant **Andrew Ustianowski, MD, PhD**, Gilead: Honoraria|Gilead: Advisory Board|GSK: Honoraria|Janssen: Honoraria|Merck: Honoraria|Merck: Advisory Board|Sanofi: Honoraria|ViiV Healthcare/GSK: Advisory Board **Jesse Thissen, MSc**, AstraZeneca: Employee|AstraZeneca: Stocks/Bonds **Seth Seegobin, PhD**, AstraZeneca: Employee|AstraZeneca: Stocks/Bonds **Rohini Beavon, PhD**, AstraZeneca: Employee|AstraZeneca: Stocks/Bonds **Kanika Dey, MSc**, AstraZeneca: Employee|AstraZeneca: Stocks/Bonds **Elizabeth J. Kelly, PhD**, AstraZeneca: Employee|AstraZeneca: Stocks/Bonds **Karen A. Near, MD**, AstraZeneca: Employee|AstraZeneca: Stocks/Bonds **Katie Streicher, PhD**, AstraZeneca: Employee|AstraZeneca: Stocks/Bonds **Alexandre Kiazand, MD**, AstraZeneca: Employee|AstraZeneca: Stocks/Bonds **Mark T. Esser, PhD**, AstraZeneca: Employee|AstraZeneca: Stocks/Bonds

